# A C-terminal motif contributes to the plasma membrane localization of *Arabidopsis* STP transporters

**DOI:** 10.1371/journal.pone.0186326

**Published:** 2017-10-13

**Authors:** Kohji Yamada, Yuriko Osakabe, Kazuko Yamaguchi-Shinozaki

**Affiliations:** 1 Graduate School of Agricultural and Life Sciences, University of Tokyo, Tokyo, Japan; 2 Graduate School of Technology, Industrial and Social Sciences, Tokushima University, Tokushima, Japan; RIKEN Biomass Engineering Program, JAPAN

## Abstract

Membrane trafficking is highly organized to maintain cellular homeostasis in any organisms. Membrane-embedded transporters are targeted to various organelles to execute appropriate partition and allocation of their substrates, such as ions or sugars. To ensure the fidelity of targeting and sorting, membrane proteins including transporters have sorting signals that specify the subcellular destination and the trafficking pathway by which the destination is to be reached. Here, we have identified a novel sorting signal (called the tri-aromatic motif) which contains three aromatic residues, two tryptophans and one histidine, for the plasma membrane localization of sugar transporters in the STP family in *Arabidopsis*. We firstly found that a C-terminal deletion disrupted the sugar uptake activity of STP1 in yeast cells. Additional deletion and mutation analyses demonstrated that the three aromatic residues in the C-terminus, conserved among all *Arabidopsis* STP transporters, were critical for sugar uptake by not only STP1 but also another STP transporter STP13. We observed that, when the tri-aromatic motif was mutated, STP1 was largely localized at the endomembrane compartments in yeast cells, indicating that this improper subcellular localization led to the loss of sugar absorption. Importantly, our further analyses uncovered that mutations of the tri-aromatic motif resulted in the endoplasmic reticulum (ER) retention of STP1 and STP13 in plant cells, suggesting that this motif is involved at the step of ER exit of STP transporters to facilitate their plasma membrane localization. Together, we here identified a novel ER export signal, and showed that appropriate sorting via the tri-aromatic motif is important for sugar absorption by STP transporters.

## Introduction

Solute transport is crucial for maintaining cellular homeostasis. Transporters are embedded in biological membranes and are required to transfer substrates across membranes at the subcellular level and the tissue level for long-distance transport [1,2]. Long-distance transport of sugars occurs via bulk flow within the phloem, and sugar transporters are responsible for loading and unloading the phloem. Because sugars have multiple functions as energy sources and signaling molecules, sugar transporter activity is increased or repressed by transcriptional and post-translational mechanisms to respond to changing environments. For example, as shown in our previous reports, the *Arabidopsis* sugar transporters *ESL1* and *STP13* are transcriptionally activated in response to environmental stress, such as cold, drought and high salinity stresses [3,4]. Tonoplast-localized ESL1 may export monosaccharides to the cytoplasm from the vacuole to increase cytoplasmic osmotic pressure [3]. Plasma membrane-localized STP13, which is expressed in root endodermal cells, retrieves monosaccharides that leak from dead epidermal and cortex cells when plants are exposed to high salinity stress [4]. Furthermore, STP13 is phosphorylated by the plasma membrane-localized receptor kinase BAK1 in leaves when the defense response is activated following the perception of microbial molecules. This phosphorylation enhances the sugar uptake activity of STP13 to restrict sugar acquisition by the pathogen [5]. In addition, the tonoplast-localized sugar transporter TST1, which was previously designated TMT1, is reported to be phosphorylated under cold conditions [6]. The mitogen-activated triple kinase-like kinase VIK1 has been identified as a kinase that phosphorylates TST1 [7]. Co-incubation of isolated vacuoles with VIK1 facilitates glucose import, suggesting that VIK1-mediated phosphorylation promotes TST1 activity [7].

In addition to transcriptional and post-translational regulation, membrane trafficking through the secretory pathway is also a critical determinant of transporter functions in physiological contexts. Transporters play a major role in nutrient uptake from soils. Radical nutrient transport from the root surface to the xylem requires at least two transport events: import into epidermal, cortical or endodermal cells and export to the xylem. For example, the boron channel NIP5;1 and the boron transporter BOR1 are predominantly involved in boron import and export, respectively, in *Arabidopsis* [8]. NIP5;1 is localized to the distal side of the plasma membrane, whereas BOR1 is localized to the proximal side. Their polarized localization is regulated by endocytosis [9] and facilitates radical boron transport from the root surface to the xylem. In addition, endocytosis-mediated BOR1 degradation occurs under high-boron conditions to prevent growth defects due to the toxicity of excess boron [10].

Membrane proteins are sorted to the membranes of various organelles after synthesis in the endoplasmic reticulum (ER). Numerous studies have shown that the first crucial step in secretory trafficking is the exit from the ER to the Golgi apparatus, which is mediated by COPII vesicles [11,12]. ER export signals reside in cytosolic regions of membrane proteins. Various ER export signals have been identified, including the di-acidic motif (DxE, x indicates any residue) of the vesicular stomatitis virus glycoprotein [13], the di-phenylalanine motif (FF) of the ER-Golgi intermediate compartment protein ERGIC-53 [14], and the di-hydrophobic and tyrosine-based motif (FVxxxY) of the *Arabidopsis* endomembrane protein EMP12 [15]. Membrane proteins are recruited into COPII vesicles through the recognition of their ER export signals. The plant potassium channel KAT1 interacts with Sec24, a component of the COPII coat, at the ER via the di-acidic motif [16]. Therefore, mutations of this motif in KAT1 resulted in reduced potassium import in cultured mammalian cells, likely due to the disruption of the normal subcellular localization of this protein [17]. Sorting to the vacuole has also been investigated. At least two transport routes to the tonoplast have been identified. The tonoplast localization of the *Arabidopsis* sucrose transporter SUC4 depends on the AP-3 adapter complex, although the localization of ESL1 and the *myo*-inositol transporter INT1 does not [18]. ESL1 and INT1 contain di-leucine motifs, which are required for the tonoplast localization, at their N-terminus and C-terminus, respectively [3,18]. When these di-leucine motifs are mutated, the proteins are mis-sorted to the plasma membrane in plant cells [3,18]. Mutated ESL1, which is located at the plasma membrane, takes up sugars from the media in tobacco BY-2 cells, but tonoplast-localized ESL1 does not [3]. In the case of transporters, the subcellular localization is coupled with the enzymatic activity to properly move substrates across membranes. Therefore, their sorting to appropriate subcellular compartments should be highly organized to maintain cellular homeostasis.

Here, we identified a novel sorting signal for the plasma membrane, the tri-aromatic motif (WxxHxxW), in the C-terminal cytoplasmic tails of *Arabidopsis* STP transporters. STP transporter variants devoid of this motif failed to complement the glucose uptake activity of multiple monosaccharide transporter-deficient yeast cells. We found that mutants lacking the tri-aromatic motif accumulated at the ER, while functional transporters were localized at the plasma membrane. These results indicate that the tri-aromatic motif promotes the plasma membrane localization of STP transporters as an ER export signal. Based on these findings, we here reported a novel ER export signal, and provided the insight in which regulation of appropriate sorting by the tri-aromatic motif is important for sugar uptake of STP transporters.

## Material and methods

### Plasmid construction

The *STP1* or *STP13* cDNAs were inserted into the pVT-102U vector along with the mGFP4 fragment [4]. C-terminal deletion mutants and tri-aromatic motif mutants were constructed as previously described [19]. In addition, each fragment was also inserted to the pRI 35S GFP vector [5] using an In-Fusion HD Cloning Kit (TaKaRa, Shiga, Japan). The primers used to construct the plasmids are listed in S1 Table.

### Yeast complementation assay

Constructs were introduced into the hexose uptake-deficient yeast strain EBY.S7 [20]. Transformants were grown on minimal media containing 2% maltose. For complementation assays, liquid-cultured yeast cells were washed with water, and their optical densities at 600 nm (OD600) were adjusted to 1.0, 0.1 and 0.01. The cells were dropped on minimal media containing 10 mM glucose and incubated at 30°C for 3 days.

### RT-PCR analysis

Total RNA was isolated from the plant samples using RNAiso reagent (TaKaRa) according to the manufacturer’s instructions, and reverse-transcribed using a PrimeScript RT reagent kit (Perfect Real Time) (TaKaRa) with an oligo(dT) primer and a random primer. The RT-PCR analysis was performed using the primers listed in S1 Table.

### Transient gene expression in *Nicotiana benthamiana*

Transient gene expression experiments in *Nicotiana benthamiana* were performed according to a previously described method [21]. *Agrobacterium tumefaciens* GV3101 strains were grown in YEP media (10 g/l bactopeptone, 10 g/l yeast extract, 5 g/l NaCl, 2 mM MgCl_2_) with appropriate antibiotics. Cultures were pelleted by centrifugation, resuspended in infiltration buffer (10 mM MgCl_2_, 10 mM MES-KOH pH 5.5) to an OD600 of 0.1, and then infiltrated into *N*. *benthamiana* leaves using a syringe. Leaves were harvested 2 days after inoculation.

### Establishment of stable transgenic *Arabidopsis* plants

pRI 35S STP1-GFP and pRI 35S STP1 (WHW/AAA)-GFP constructs were transformed in *Arabidopsis thaliana* (Col-0) via *Agrobacterium-*mediated transformation.

## Results

### C-terminal deletion disrupts sugar uptake by STP1

**Fig 1:**
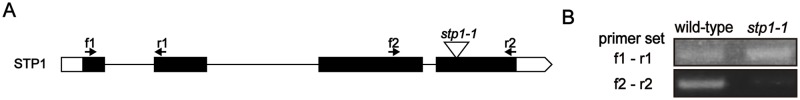
The 5’ fragment of *STP1* that was expressed in *stp1-1* plants. (A) A schematic view of the T-DNA inserted into the *stp1-1* mutant and positions of the primers used in (B). (B) RT-PCR analysis of *STP1* expression in wild-type and *stp1-1* plants. Similar results were obtained in three independent experiments.

As shown in our previous study, *stp1-1* plants, which contain a T-DNA insert in the *STP1* locus of the *Arabidopsis* genome, exhibited reduced sugar uptake [4, 5]. However, we wondered whether the STP1 protein in *stp1-1* plants is surely non-functional because the T-DNA is inserted at the 3’-end of the *STP1* coding sequence (Fig 1A). Previously, the accumulation of the full-length *STP1* mRNA was not detected in *stp1-1* plants [4]. The abnormal *STP1* mRNA containing the T-DNA insertion may be degraded immediately, causing the loss-of-STP1 phenotype in *stp1-1* plants. We first attempted to amplify 5’ and 3’ fragments of *STP1* in the *stp1-1* plants to investigate this hypothesis. We observed comparable accumulation of the 5’ fragment of *STP1* in wild-type (WT) and *stp1-1* plants, whereas the 3’ fragment of *STP1*, the region containing the T-DNA insert, was not detected in *stp1-1* plants (Fig 1B). Thus, these data suggested that the C-terminally truncated STP1 protein is expressed in *stp1-1* plants but does not function in sugar absorption.

**Fig 2:**
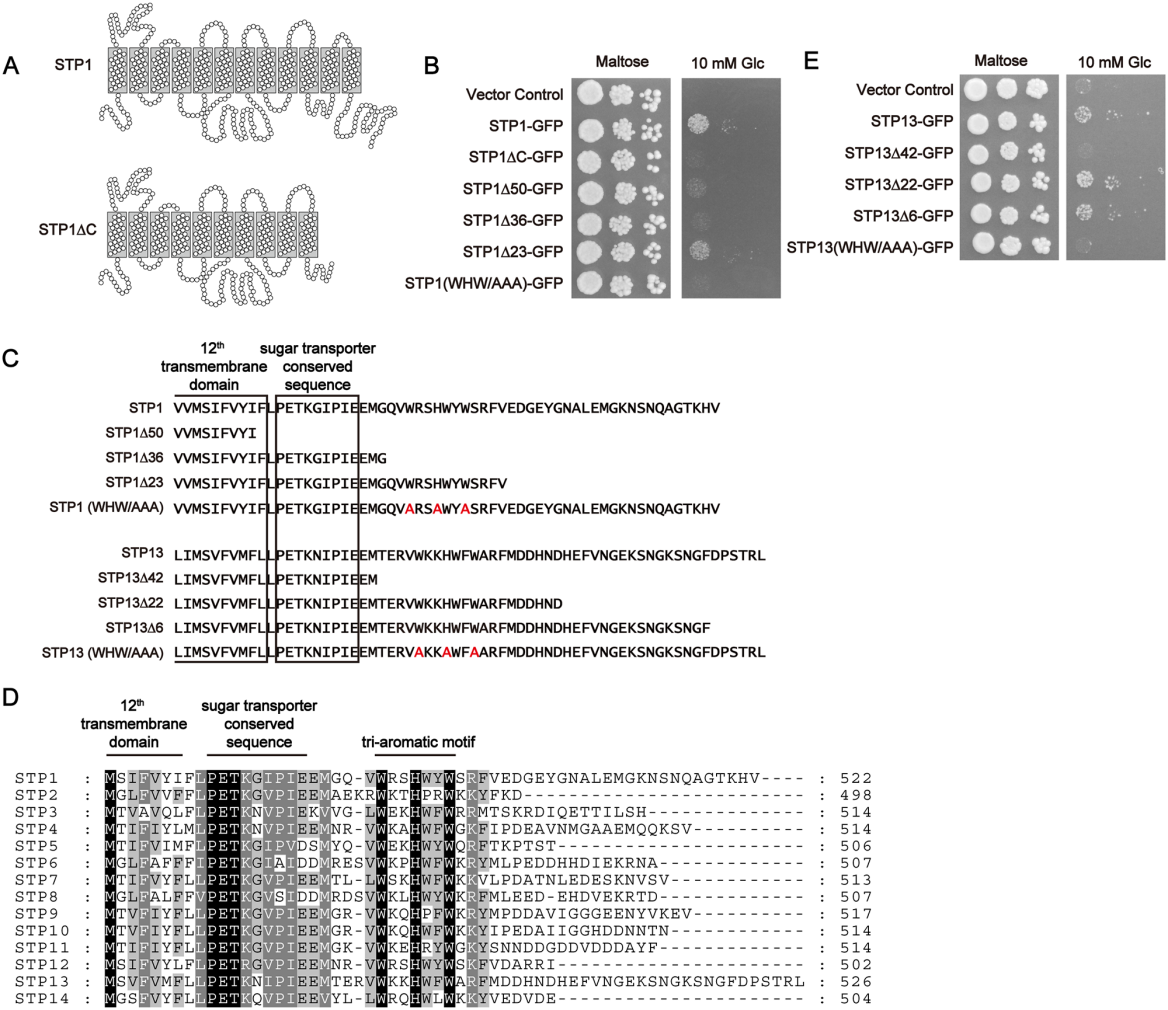
The C-terminal aromatic residues are required for the glucose uptake by STP1 and STP13. (A) Schematic view of the predicted structure of STP1. STP1ΔC, a C-terminally truncated variant, is expected to be expressed in *stp1-1* plants. Circles and gray rectangles indicate amino acid residues and transmembrane domains, respectively. (B and E) Complementation assays for glucose uptake in yeast cells. Transformed yeast cells were grown on medium containing 2% maltose (control) or 10 mM glucose for 3 days. (C) Sequences of the C-terminally truncated or amino acid-substituted STP1 and STP13 mutants. Substituted amino acids are highlighted in red. (D) Alignment of the C-terminal regions of *Arabidopsis* STP transporters. Similar results were obtained in three independent experiments.

We expressed the STP1ΔC protein (Fig 2A), which lacked the C-terminal region beginning at the 11^th^ transmembrane domain, in yeast cells to investigate the functionality of the C-terminally truncated STP1 protein. A similarly truncated STP1 variant is expected to be expressed in *stp1-1* plants; however, the STP1 protein expressed in *stp1-1* plants should contain several additional residues from the T-DNA sequence compared with STP1ΔC. The EBY.S7 yeast strain used in this study is not able to grow on media containing glucose as the sole carbon source because it lacks multiple monosaccharide transporters [20]. Consistent with our previous report [4], the introduction of STP1-GFP complemented yeast growth on glucose media (Fig 2B). However, the introduction of STP1ΔC-GFP did not affect yeast growth (Fig 2B). These results suggested that the C-terminally truncated STP1 protein expressed in *spt1-1* plants is not functional for sugar absorption.

### The tri-aromatic motif (WxxHxxW) is required for sugar uptake by STP transporters

These findings prompted us to explore the region required for sugar uptake by STP1. First, we hypothesized that the lack of two transmembrane domains disrupted the three-dimensional structure of STP1, resulting in the loss of the sugar transporter activity of STP1. Thus, we established the STP1Δ50 variant, which lacks only the C-terminal tail (Fig 2C). However, the introduction of STP1Δ50-GFP also failed to complement the growth of monosaccharide transporter-deficient yeast cells on glucose media (Fig 2B). We next generated the STP1Δ36 variant to investigate the importance of the sugar transporter conserved sequence in the C-terminal tail (Fig 2C), but its introduction did not promote yeast growth either (Fig 2B). However, the STP1Δ23 variant complemented yeast growth on glucose media (Fig 2B), indicating that the 13 amino acids from STP1Δ23 but not STP1Δ36 are important for sugar absorption. An alignment analysis of the C-terminal tails of 14 *Arabidopsis* STP transporters revealed that this region is highly conserved among *Arabidopsis* STP transporters (Fig 2D). Because we identified three completely conserved aromatic residues, two tryptophan residues (W) and one histidine residue (H), in this region, we designated this sequence (WxxHxxW) the tri-aromatic motif in this study (Fig 2D).

To further investigate the importance of the tri-aromatic motif in other STP transporters, we established the STP13Δ42 variant which does not contain the tri-aromatic motif (Fig 2C). The introduction of STP13Δ42-GFP did not promote the yeast growth, whereas the introduction of STP13-GFP or the GFP-fused STP13 variants containing the tri-aromatic motif allowed the yeast cells to grow on glucose media (Fig 2E).

Next, we established STP1 (WHW/AAA) and STP13 (WHW/AAA) variants, in which the W and H residues in the tri-aromatic motif were substituted with alanine (A) residues, to investigate the importance of these three residues in the tri-aromatic motif (Fig 2C). As expected, we did not observe an increase in yeast growth when STP1 (WHW/AAA)-GFP or STP13 (WHW/AAA)-GFP was introduced (Fig 2B and 2E). Taken together, these data demonstrated that the three aromatic resides in the tri-aromatic motif are critical for sugar uptake by STP transporters.

### The tri-aromatic motif functions as an ER export signal of STP transporters

**Fig 3:**
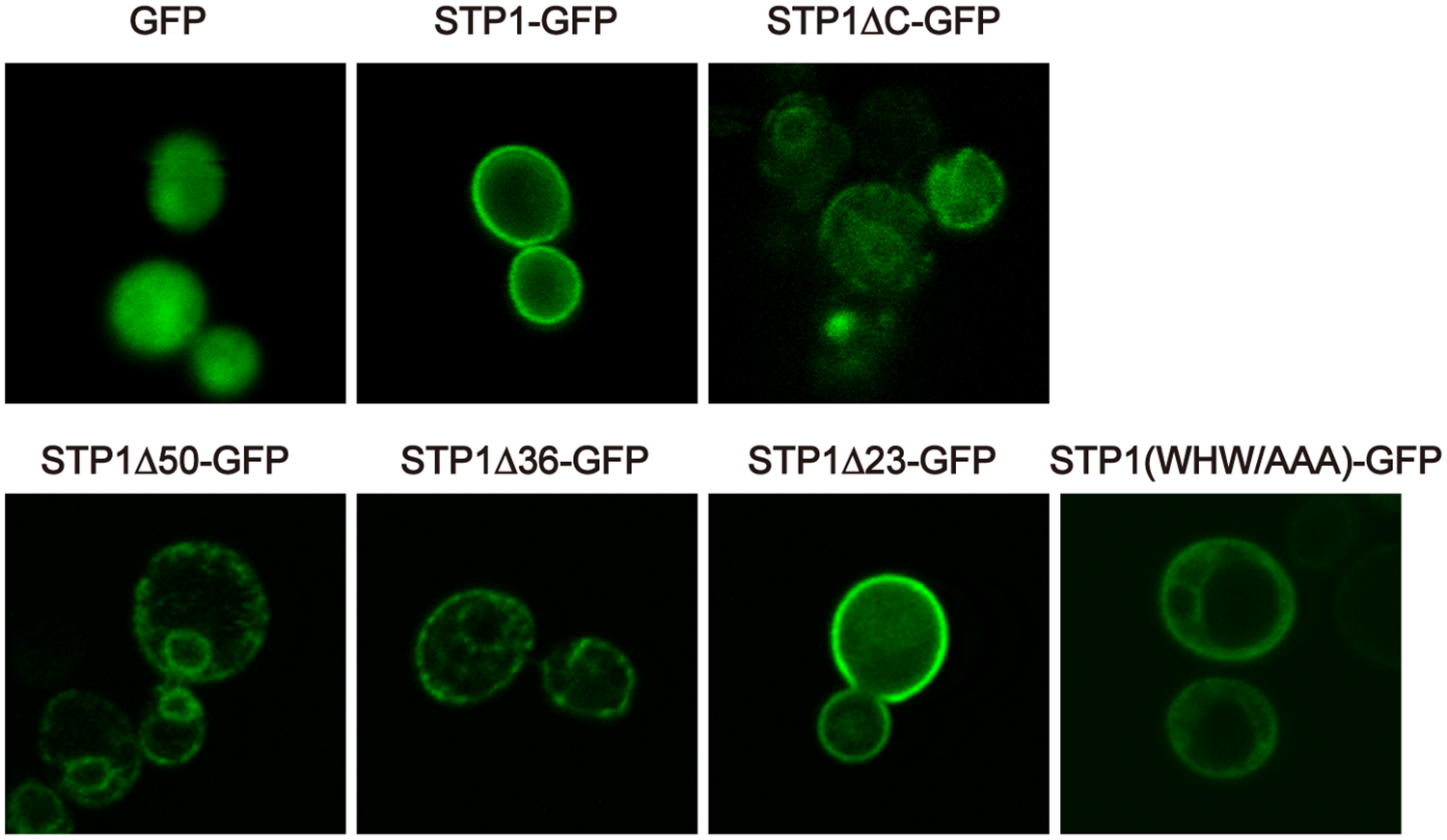
The tri-aromatic motif is important for STP1 trafficking to the plasma membrane in yeast cells. GFP signals were observed in yeast cells expressing STP1-GFP variants. Similar results were obtained in three independent experiments.

We explored how the tri-aromatic motif affects sugar uptake activities of STP transporters. We observed GFP signals from a series of STP1-GFP mutants in yeast cells to determine whether the C-terminal deletions affect the behavior of STP1. GFP signals from the non-functional variants STP1ΔC-GFP, STP1Δ50-GFP and STP1Δ36-GFP were observed in the endomembrane compartments in yeast cells, but the fluorescent signals from the functional STP1Δ23-GFP variant and STP1-GFP were detected at the plasma membrane (Fig 3). Furthermore, we also detected the endomembrane localization of STP1 (WHW/AAA)-GFP in yeast cells (Fig 3). Thus, their localization at the endomembrane compartments likely resulted in the loss of sugar absorption from the extracellular spaces in yeast cells (Fig 2B).

**Fig 4:**
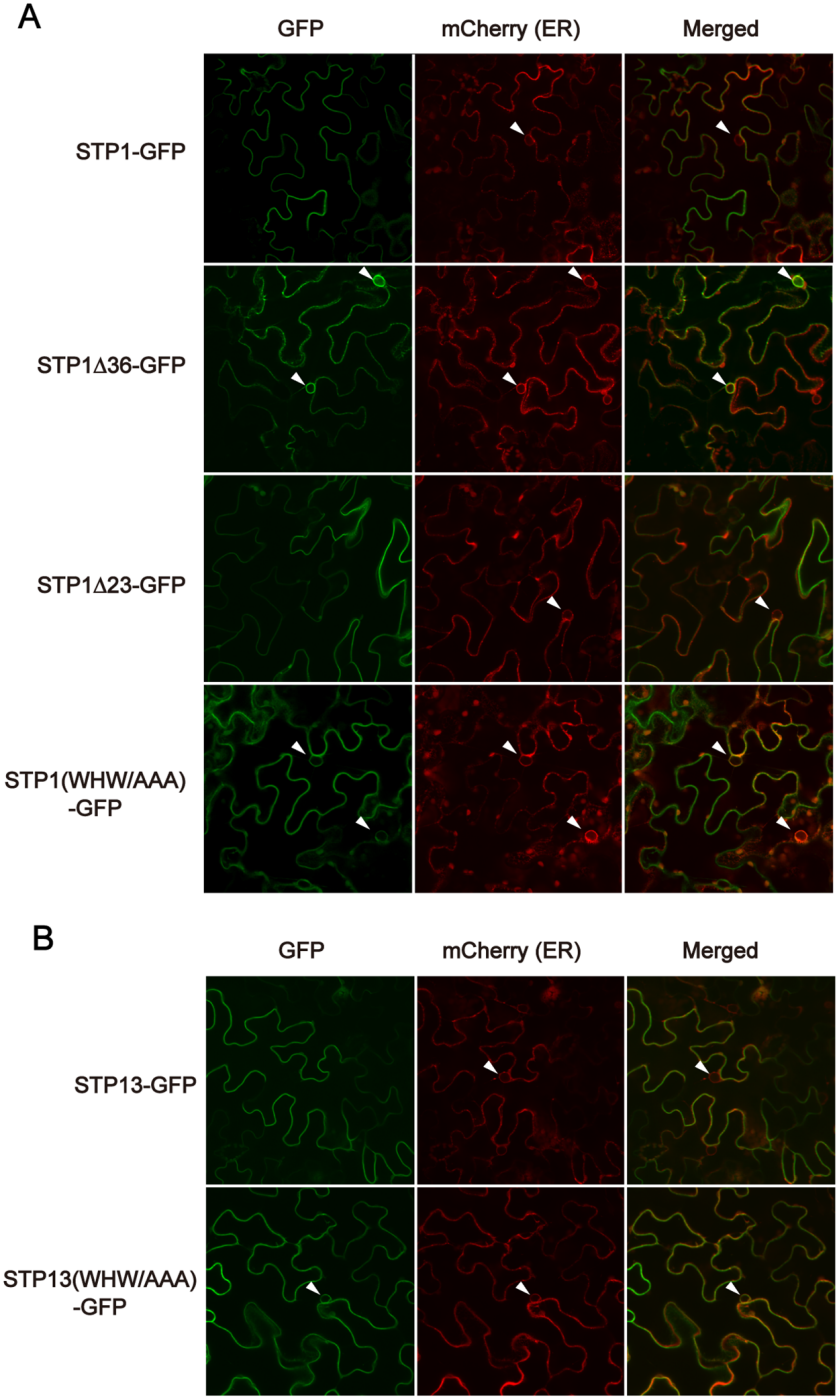
Disruption of the tri-aromatic motif causes ER retention of STP1 and STP13 in *N*. *benthamiana* leaves. (A and B) GFP and mCherry signals were observed for STP1 and STP13 variants and an ER marker, respectively, expressed in *N*. *benthamiana* leaves. Arrowheads indicate perinuclear regions. Similar results were obtained in three independent experiments.

We next introduced these STP1 variants into *Nicotiana benthamiana* leaves via agro-infiltration to determine whether the tri-aromatic motif also contributed to the plasma membrane localization of STP transporters in plant cells. GFP signals from the non-functional variants STP1Δ36-GFP and STP1 (WHW/AAA)-GFP were strongly observed at the perinuclear regions. We here noticed that GFP signals from the non-functional STP1 variants were also detected at the perinuclear regions of yeast cells (Fig 3). This fluorescent pattern suggested the ER localization of non-functional STP1 variants. Therefore, we next co-expressed an mCherry-fused ER marker protein [22] with a series of GFP-fused STP1 variants in *N*. *benthamiana* to investigate their ER localization. As expected, the mCherry signal was also observed at the perinuclear region, and merged with GFP signals from STP1Δ36-GFP and STP1 (WHW/AAA)-GFP (Fig 4A), indicating that these STP1 variants were localized at the ER. In contrast, GFP signals from STP1-GFP and the functional variant STP1Δ23-GFP were mainly detected at the cellular peripheral regions, indicating their plasma membrane localization (Fig 4A). Consistent with the above-described results, the GFP signal from STP13 (WHW/AAA)-GFP, but not STP13-GFP, was also strongly observed at the perinuclear regions in *N*. *benthamiana* leaves (Fig 4B). Although these results showed that the loss of the tri-aromatic motif caused ER retention of STP1 and STP13, their variants devoid of the tri-aromatic motif also appeared to be localized at the cellular peripheral regions. ln fact, we found that the GFP signal from STP1 (AAA/WHW)-GFP was merged with fluorescence of not only the ER marker but also the plasma membrane-staining dye FM4-64 (S1 Fig). It is indeed technically difficult to discriminate the plasma membrane and the ER at the cellular peripheral regions in leaf epidermal cells whose cytoplasmic volumes are very limited due to large central vacuoles. Therefore, we here cannot exclude the possibility that a portion of STP1 (AAA/WHW)-GFP was sorted to the plasma membrane.

**Fig 5:**
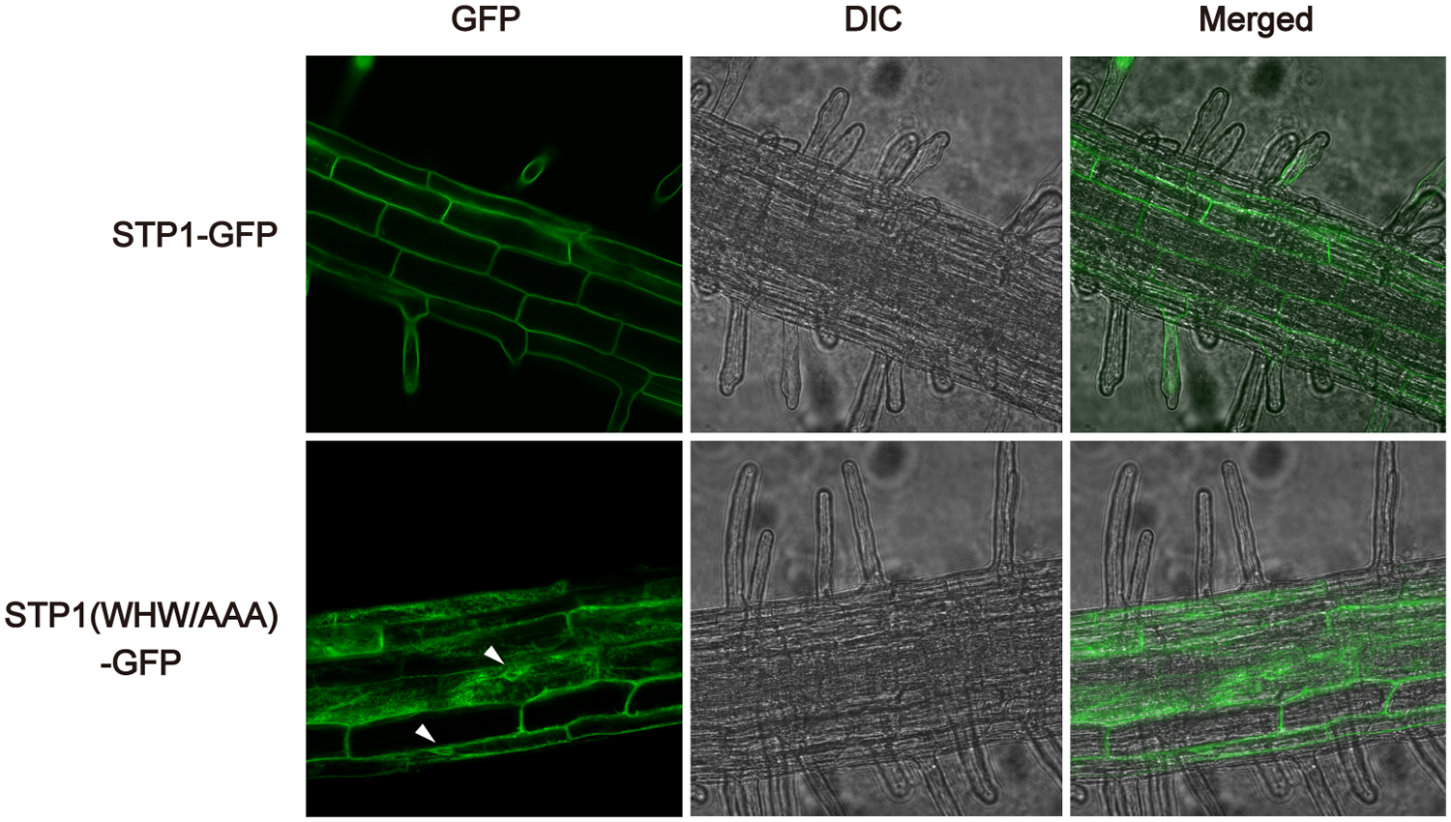
Disruption of the tri-aromatic motif causes ER retention of STP1 and STP13 in roots of stable transgenic *Arabidopsis* plants. GFP signals were observed for STP1-GFP and STP1 (WHW/AAA)-GFP in root cells of 10-day-old *Arabidopsis* plants. Arrowheads indicate perinuclear regions. Similar results were obtained in three independent experiments.

We further analyzed this observation using stable transgenic *Arabidopsis* plants. Because expression levels of genes transiently introduced to cells often exceed those stably expressed in transgenic plants, transient expression analysis sometimes causes mis-localization of protein. In transgenic *Arabidopsis* roots, GFP fluorescence from STP1 (WHW/AAA)-GFP was clearly detected in perinuclear regions and reticular structures resembling ER networks, whereas the fluorescent signal from STP1-GFP was observed at the plasma membrane (Fig 5), showing that STP1 (WHW/AAA)-GFP was localized at the ER in *Arabidopsis*. Taken all data together, we inferred that the tri-aromatic motif is involved at the step of ER exit of STP transporters as an ER export signal to facilitate their plasma membrane localization.

### The tri-aromatic motif is conserved in STP transporters of various plant species

**Fig 6:**
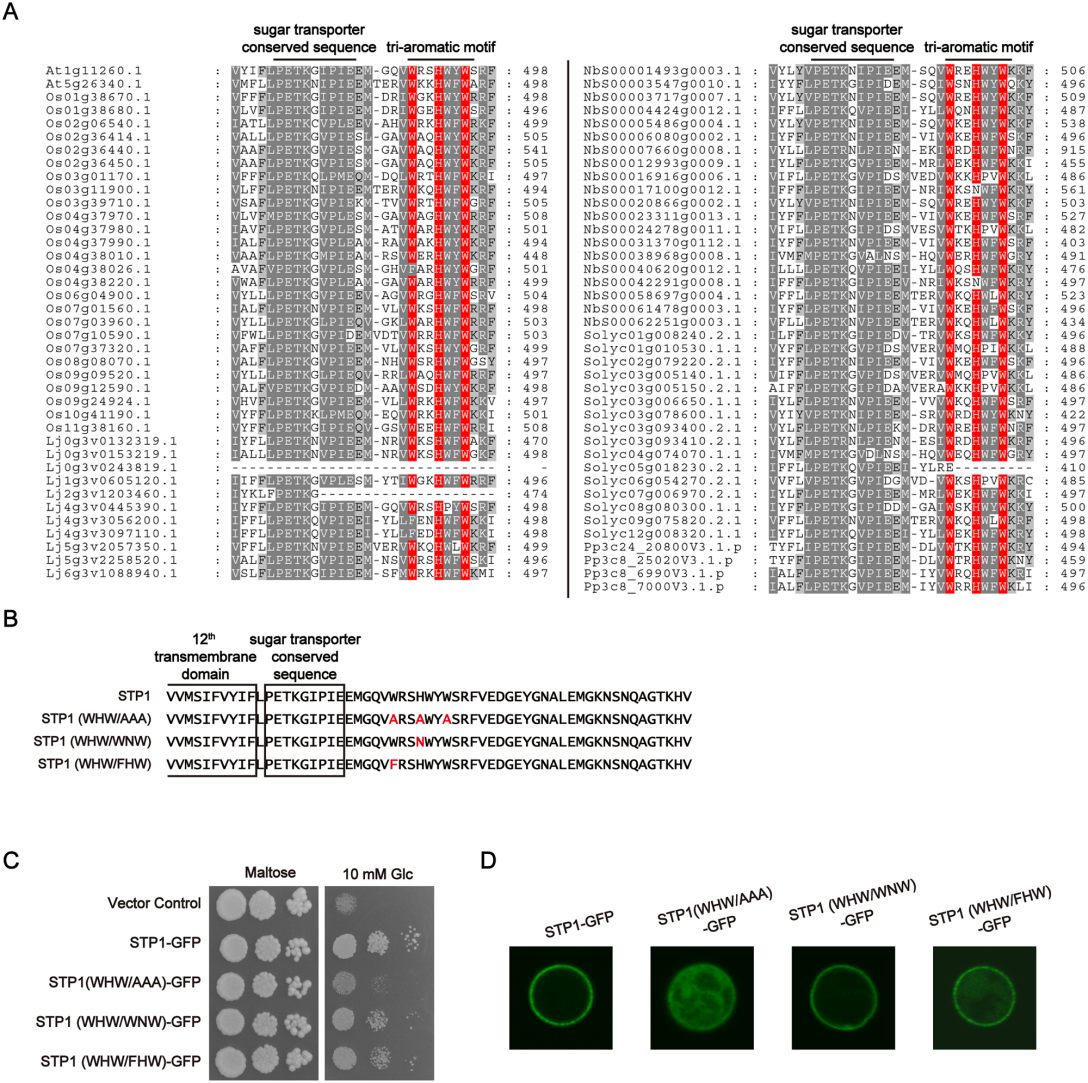
The tri-aromatic motif is conserved in STP transporters of various plant species. (A) Alignment of the tri-aromatic motifs of STP transporters from various plant species. At1g11260.1 and At5g26340.1 indicate STP1 and STP13 of *Arabidopsis*, respectively. Conserved aromatic residues in the tri-aromatic motif are highlighted in red. Os, Lj, Nb, Solyc, and Pp indicate *Oryza sativa*, *Lotus japonicas*, *Nicotiana benthamiana*, *Solanum lycoperisucum* and *Physcomitrella patens*, respectively. (B) Sequences of the amino acid-substituted STP1 mutants. Substituted amino acids are highlighted in red. (C) Complementation assays for glucose uptake in yeast cells. Transformed yeast cells were grown on medium containing 2% maltose (control) or 10 mM glucose for 3 days. (D) GFP signals were observed in yeast cells expressing STP1-GFP variants. Similar results were obtained in three independent experiments.

Because the tri-aromatic motif was observed in all *Arabidopsis* STP transporters (Fig 2D), we next investigated whether this motif occurs among other plant STP transporters. Our analysis showed that the tri-aromatic motif is highly conserved in STP transporters of the various plant species including a moss (Fig 6A), further suggesting its importance in STP transporters. However, while this motif is completely conserved in STP transporters of *Arabidopsis* and *Physcimitrelle patens*, STP transporters whose tri-aromatic motifs are mutated or deleted are found in *Oryza sativa*, *Lotus japonicus*, *N*. *benthamiana* and *Solanum lycoperisucum*. In NbS00017100g0012.1 and NbS00042291g0008.1, the histidine residue of the tri-aromatic motif is substituted with asparagine residue (N). In Os04g38026.1, Lj4g3v3056200.1 and Lj4g3097110.1, the first tryptophan residue of the tri-aromatic motif is substituted with phenylalanine residue. To investigate whether these substitutions affect sugar uptake activities of STP transporters, we generated STP1 (WHW/WNW) and STP1 (WHW/FHW) variants, the histidine and the first tryptophan residue of which was substituted with phenylalanine and asparagine residue, respectively (Fig 6B). The introduction of STP1 (WHW/WNW)-GFP and STP1 (WHW/FHW)-GFP complemented the yeast growth on glucose media. In addition, their GFP signals were observed at the plasma membrane in yeast cells (Fig 6D). We here noted that these STP1 variant cells seemed to grow to a slightly lesser extent with STP1-GFP cells on glucose media (Fig 6C) and florescence of STP1 (WHW/FHW)-GFP appeared to be patchly distributed at plasma membrane (Fig 6D), which suggested the possibility that these substations somewhat affect STP1 behavior. Nonetheless, our data indicated that STP transporters, which even have these substitutions, are also functional for sugar absorption.

## Discussion

Here, we showed that a novel sorting signal, the tri-aromatic motif (WxxHxxW), acts as an ER export signal to promote the plasma membrane localization of STP1 and STP13. Although the tri-aromatic motif is highly conserved in STP transporters in various plant species, natural variations in this motif occur in some STP transporters (Fig 6A). Our data indicated that STP transporters, which have these substitutions, are functional enough to complement the sugar uptake activity of the multiple sugar transporter-deficient yeast cells (Fig 6C). Such functional conservation may also support the importance of the tri-aromatic motif in STP transporters. The correct trafficking of transporters is indeed critical for their physiological functions. For example, PHF1, a plant-specific SEC12-related gene, governs the sorting of the phosphate transporter PHT1;1 from the ER to the plasma membrane in *Arabidopsis* [23]. Thus, the loss of PHF1 causes the ER retention of PHT1;1, and *phf1* plants become sensitive to low-phosphate conditions [23]. Additionally, interfering with clathrin function results in defects in endocytosis and the polar localization of the auxin transporter PIN1, perturbing the normal auxin distribution in *Arabidopsis* [24]. Although we demonstrated that the tri-aromatic motif affects the subcellular localization of STP1 and STP13, we were here not able to address whether it also contributes to their enzymatic properties. Since the improper localization of transporters eventually disrupts their substrate movements across the membranes, it is difficult to uncouple the subcellular localization and the enzymatic activity in our experimental systems. However, our data suggested the possibility that STP1 (WHW/AAA)-GFP was partially sorted to the plasma membrane in *N*. *benthamiana* leaves (S1 Fig) although STP1 (WHW/AAA)-GFP was a non-functional transporter in yeast cells (Fig 2B). Whereas further investigation is required to determine more detailed roles of the tri-aromatic motif in STP transporters, it implies that this motif is involved in the sugar uptake activity of STP1.

Sorting signals reside in cytoplasmic regions of multipass transmembrane proteins, such as transporters. We identified the tri-aromatic motif as a novel sorting signal in the C-terminal cytoplasmic tails of STP transporters. In particular, the tri-aromatic motif is important when STP transporters are exported from the ER. The ER protein quality control system recognizes incorrect folding proteins and retains these proteins in the ER. Therefore, mutations or deletions which we here introduced to STP1 and STP13 might result in their ER localization due to improper protein folding. Although we cannot exclude this possibility, we have considered that mutations of only three residues in cytoplasmic tails, not in transmembrane domains, may not significantly affect their structure. Therefore, based on our data, we postulate that the tri-aromatic motif functions as an ER export signal promoting the plasma membrane localization of STP transporters. In addition to ER exit, membrane trafficking to the plasma membrane is highly regulated by the Golgi/*trans*-Golgi network. The tri-aromatic motif may also be involved in post-ER trafficking to the plasma membrane. GFP fluorescence patterns of STP1 (AAA/WHW)-GFP and STP1Δ36-GFP, which partially retained and completely lost the tri-aromatic motif sequence, respectively, seemed to be different in yeast cells (Fig 3). We noticed that several amino acids adjacent to the three aromatic residues are also highly conserved in the tri-aromatic motif (Figs 2D and 6). Collectively, these findings imply that these conserved residues play a role in the subcellular localization of STP1. In the case of *Arabidopsis* EMP12 which is localized in the Golgi apparatus, it has two sorting signals for ER export and Golgi retention in the C-terminus [15].

ER export signals are categorized into two major groups, the acidic motif and the hydrophobic motif [12]. Hydrophobic motifs of ER export signals often contain aromatic amino acids, like the tri-aromatic motif. Protein trafficking between the ER and the Golgi apparatus is mediated by two distinct coated vesicles, COPII and COPI, which regulate anterograde and retrograde transport, respectively [11,12]. ER export signals in membrane proteins are recognized and bound by Sar1-Sec23-Sec24 prebudding complexes for inclusion in COPII vesicles [12]. For example, the aromatic residue-containing FVxxxY motif of the *Arabidopsis* EMP12 protein binds to Sec24 *in vitro* [15]. The tri-aromatic motif may also interact with COP II prebudding complexes, although further investigations are needed to determine the mechanism by which the tri-aromatic motif contributes to the proper sorting of STP transporters. Notably, the tri-aromatic motif was functional in yeast and plant cells in this study, indicating that the secretory pathway mediated by the tri-aromatic motif is conserved in different organisms. Indeed, a similar motif (FxxxFxxxF) has been reported to play a role in ER exit of the dopamine receptor D1 in cultured mammalian cells [25]. The ER membrane-associated protein DRiP78 was reported to bind to this motif and regulate D1 trafficking via a process that remains elusive [25]. Because DRiP78 is also conserved in *Arabidopsis* [25], it may contribute to protein sorting through the pathway mediated by the tri-aromatic motif.

This study revealed that a novel sorting signal, which is highly conserved among STP transporters in various plant species, is important for STP function. In conclusion, the tri-aromatic motif promotes the plasma membrane localization of STP transporters as an ER export signal, and our findings provide insights into the importance of appropriate subcellular localization for the physiological functions of transporters.

## Supporting information

S1 Fig Co-localization analysis of STP1 variant with the plasma membrane-staining dye FM4-64 in *N*.*benthamiana*. Fluorescent signals were observed from STP1 variants and the plasma membrane-staining dye FM4-64 in *N*. *benthamiana* leaves. Arrowheads indicate perinuclear regions.
(TIF)

S1 Table Primers used in this study. (TIF)
