# Supplementary figures and images for: A C-terminal motif contributes to the plasma membrane localization of Arabidopsis STP transporters
Source: PLoS One. 2017 Oct 13;12(10):e0186326. doi: 10.1371/journal.pone.0186326 (PMC5640241; doi:10.1371/journal.pone.0186326)

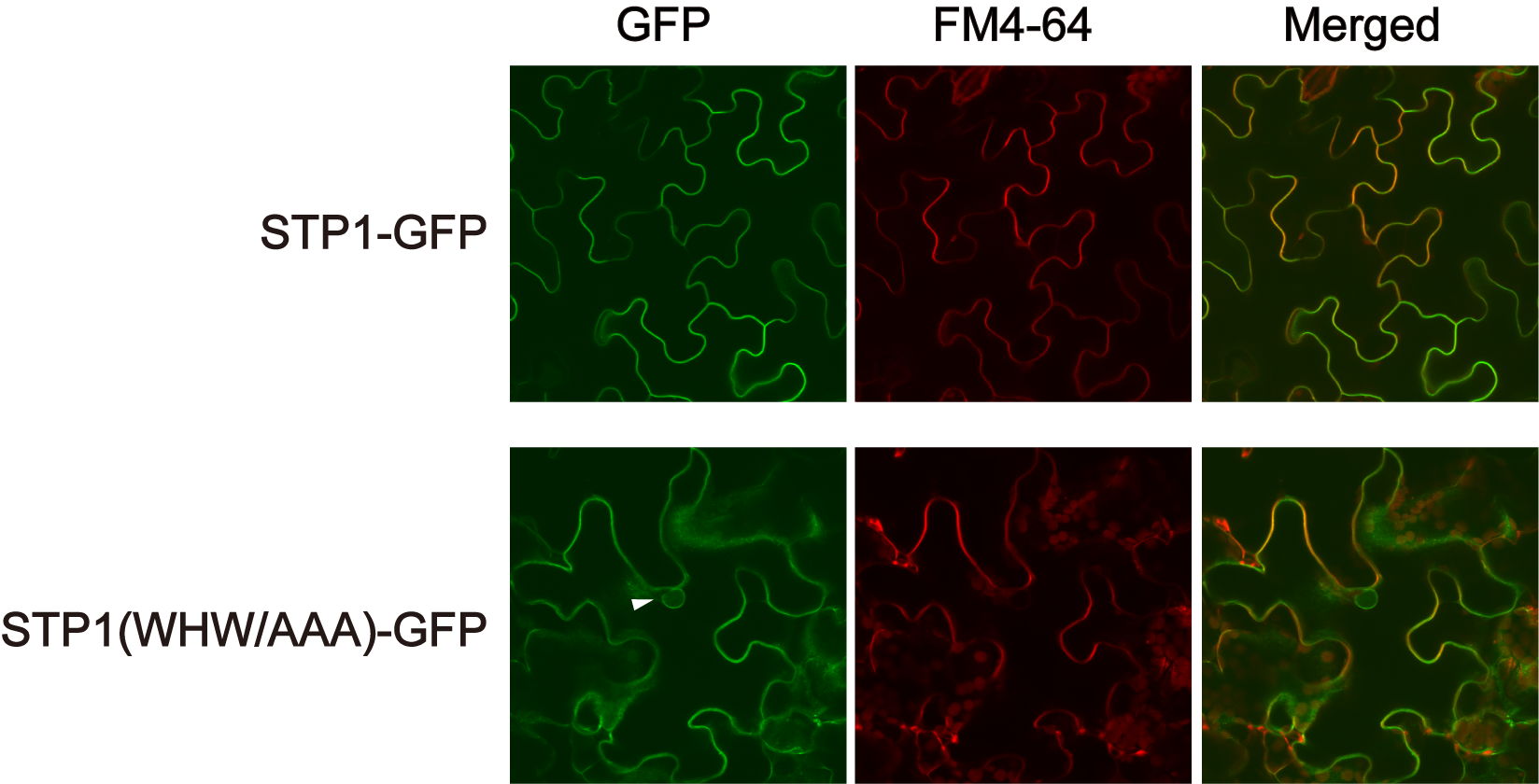

Supplement: S1 Fig — Fluorescent signals were observed from STP1 variants and the plasma membrane-staining dye FM4-64 in N. benthamiana leaves. Arrowheads indicate perinuclear regions. (TIF) [file pone.0186326.s001.tif]

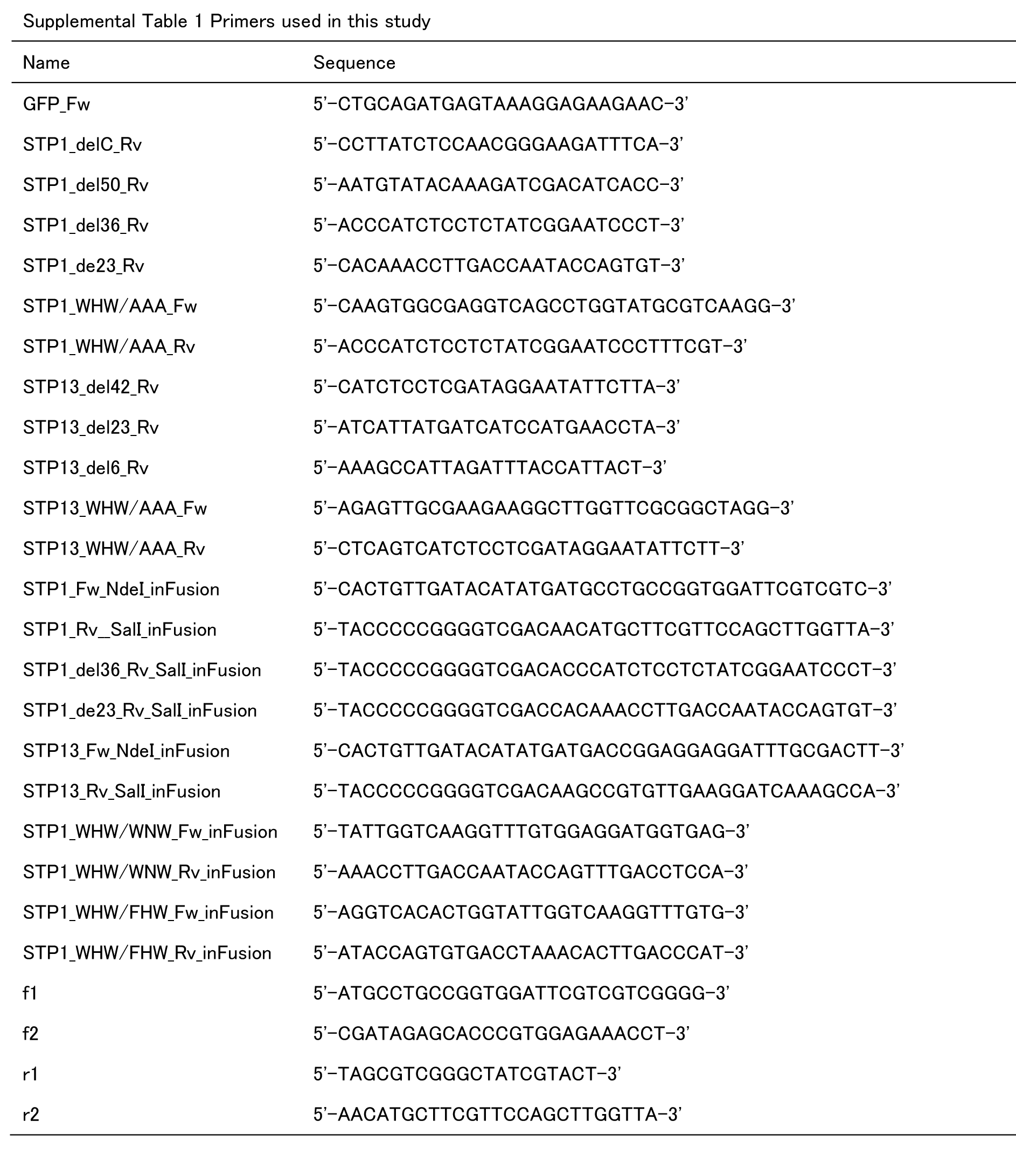

Supplement: S1 Table — (TIF) [file pone.0186326.s002.tif]
